# Inferior Vena Cava Filter Thrombosis in the Postoperative Neurosurgical Setting: Case Report and Review of the Literature

**DOI:** 10.7759/cureus.529

**Published:** 2016-03-10

**Authors:** Daniel Loriaux, Mary In-Ping Huang Cobb, Ali Zomorodi, Fernando Gonzalez, Tony P Smith, Shivanand P Lad

**Affiliations:** 1 School of Medicine, Duke University Medical Center, Durham NC; 2 Neurosurgery, Duke University Medical Center, Durham NC; 3 Neurosurgery , Duke University Medical Center, Durham NC; 4 Radiology, Duke University Medical Center, Durham NC

**Keywords:** ivc filter, postoperative complications, neurosurgery

## Abstract

There are no definitive treatment guidelines for caval-filter thrombosis in the postoperative setting. Clinical management for partial or complete postoperative inferior vena cava (IVC) occlusion relies solely on expert opinion, anecdotal evidence, and small clinical trials. As such, the primary objective of the present report is to offer a complex case of extensive IVC filter occlusion in a neurosurgical patient with past medical history significant for protein C deficiency. The presentation, unique radiological findings, management, and outcome will be discussed. No similar cases of massive IVC-occlusive disease in a thrombophilic patient early in the postoperative course following neurosurgical intervention are documented in the medical literature.

## Introduction

Over recent years, the use of inferior vena cava (IVC) filters for primary thromboprophylaxis in surgical patients has substantially increased [1]. Currently, there are only two accepted indications for IVC filter placement: (1) patients with thromboembolism and a coexisting absolute contraindication for therapeutic anticoagulation, and (2) patients with recurrent pulmonary embolism (PE) despite therapeutic anticoagulation [2-4]. Outside of these two indications, the use of IVC filters for venous thromboembolism (VTE) prophylaxis is controversial in the literature and inconsistent in practice [5]. IVC filter thrombosis is a challenging diagnosis in the postoperative setting as the constellation of symptoms suggestive of IVC occlusion includes oliguria, edema, and pain, which are all nonspecific and expected physiologic responses to surgical intervention [6-8]. Thus, efficient diagnosis and treatment of IVC filter occlusion postoperatively are dependent upon close monitoring and a low threshold for acquiring CT abdominal imaging.

Despite dramatically increased use of IVC filters in recent years, the indications, safety, and effectiveness of the filter (especially in the perioperative setting) remain understudied and controversial [2, 9-12]. Recent neurosurgical intervention is regarded as an absolute contraindication to pharmacologic anticoagulation due to the devastating consequences of hematoma formation should it arise within the intracranial cavity or spinal canal, which are both ‘closed’ and unforgiving anatomical compartments [13]. Non-neurosurgical procedures, by contrast, are regarded only as relative contraindications to postoperative therapeutic anticoagulation. Therefore, the presentation and management of a neurosurgical patient with congenital thrombophilia and IVC occlusion is particularly complex and lacks even Level IV evidence in the medical literature. This report is, to our knowledge, the only case of extensive IVC filter occlusion in a postoperative patient following neurosurgical intervention available in the literature.

## Case presentation

A 57-year-old male with past medical history significant for protein C deficiency presented to the authors' practice with severe trigeminal neuralgia refractory to medical management. After seven years of conservative therapy, the patient complained of worsening neuralgic pain in a left V3 distribution complicated by neurocognitive side effects secondary to his high-dose antineuralgic medications. With progressing symptoms and a side effect profile that limited any further escalation in medical management, the patient opted for operative intervention. Preoperative prophylactic IVC filter placement was arranged since the patient would have to stop his anticoagulation in the perioperative period. A hematology consultation was obtained preoperatively for postoperative co-management.​ Informed patient consent was obtained for this study.

**Figure 1:**
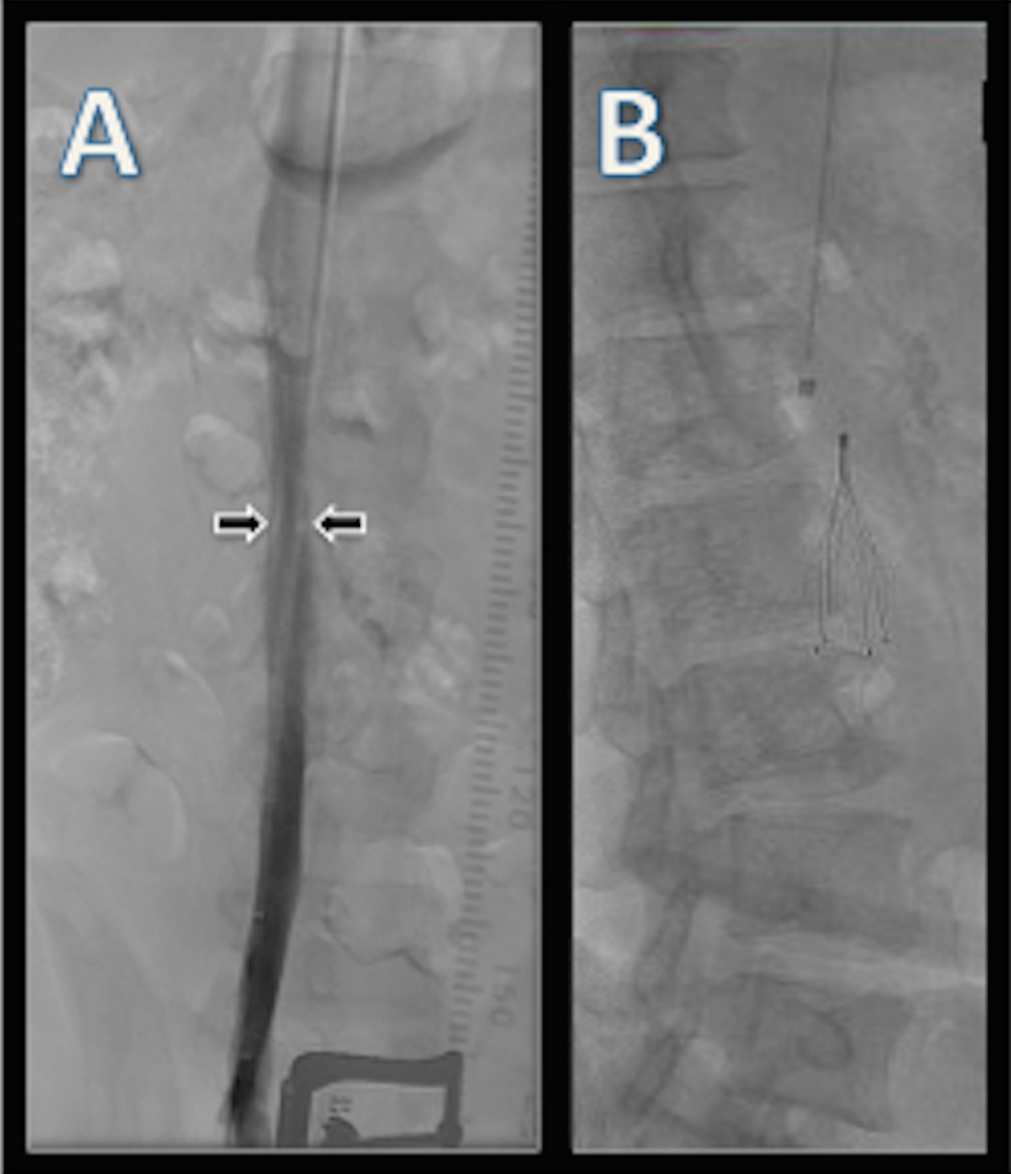
Normal preoperative inferior venacavogram Image (A) shows post-contrast, normal-caliber, widely patent IVC prior to deployment of the IVC filter. Image (B) displays the Celect retrievable IVC filter following initial placement. The filter is shown below the renal veins and with no evidence of tilting, stenosis, or pre-existing thrombus.

An IVC filter was placed on the day prior to left-sided microvascular decompression surgery. Venous access was obtained under sonographic guidance via the right internal jugular vein. A retrievable Celect IVC (Cook Celect®, Cook Medical INC., IN, USA) filter was deployed below the renal veins. Spot images of filter placement were acquired to achieve accurate positioning. An inferior venacavogram was obtained during the procedure and confirmed normal caliber IVC with no evidence of stenosis or pre-existing thrombus (Figure 1).

With the IVC filter in place, the patient was then taken to the operating room to undergo microvascular decompression. A lumbar drain was placed and a left retromastoid craniotomy was performed. The cerebellum was gently retracted inferolaterally to allow visualization of the trigeminal nerve. A large, tortuous loop of the superior cerebellar artery was identified extending from the basilar artery, coursing beneath the fifth cranial nerve, and looping back up superiorly to the entry zone of the trigeminal nerve root. Teflon pledgets were inserted between the nerve and vessel to achieve adequate decompression. The surgery itself was without complication.​

Postoperatively, the patient reported complete resolution of the trigeminal neuralgia. After an uncomplicated two-day hospital course, he was discharged home on an enoxaparin bridge to warfarin with hematology follow-up scheduled for continued management. Thirteen days postoperatively, the patient noticed acute-onset lumbar back pain with associated radicular pain. He endorsed painful episodes that emanated from a point of origin in his lumbar spine, traveled bilaterally down the posterior aspect of his legs, and terminated at mid-calf. These symptoms progressed for several days until postoperative day 16, when he presented to the Emergency Department with incapacitating pain. The patient was unable to stand or ambulate independently. He had 2+ pitting edema of both ankles and calves. An ultrasound revealed occlusive thrombosis in the right greater saphenous vein, right common femoral, and right femoral vein. A nonocclusive thrombus was found in the right popliteal vein. The patient’s INR upon arrival to the ED was subtherapeutic at 1.3.

**Figure 2:**
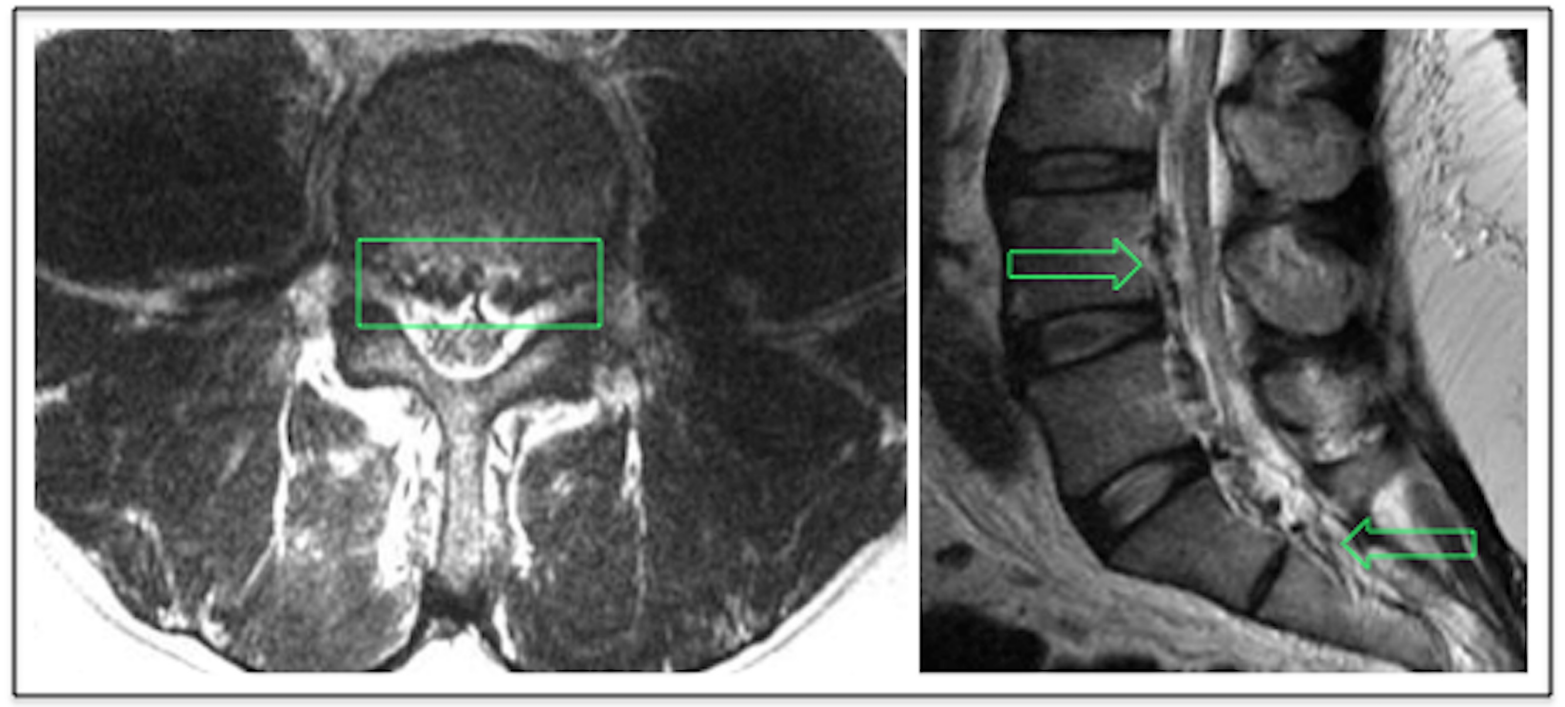
Postoperative lumbar MR imaging The left pane displays the MRI T2 axial section showing prominent flow voids (boxed) at the level of L4. The right pane displays an MRI T2 sagittal section with prominent ventral epidural enhancement (arrows) that is consistent with engorged vessels. This is shown causing narrowing of the thecal sac. This effect is most severe at the L4 vertebral level (boxed region of left image).

**Figure 3:**
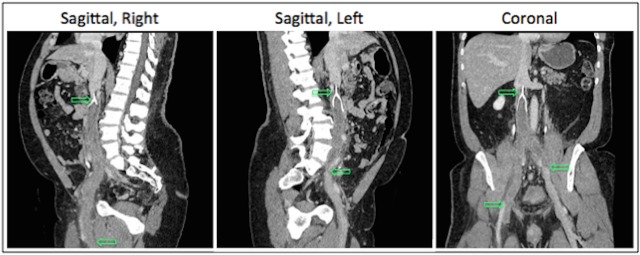
Postoperative abdominal CT imaging Sagittal and coronal CT imaging illustrating margins (arrows) of extensive, occlusive thrombosis of the inferior vena cava. The thrombus begins slightly proximal to the IVC filter and extends distally to the level of the confluence of the internal and external iliac veins on the left and out of the field of view on the right.

Magnetic resonance (MR) imaging of the lumbar spine was ordered to rule out hematoma or abscess formation as the underlying cause of the patient's myelopathic symptoms. The MR imaging revealed enhancing tissue within the ventral epidural space, extending from the level of L3 through the sacrum. Additionally, prominent flow voids could be seen within the lateral recesses on T2-weighted sequences, consistent with engorged epidural venous plexuses. The engorged collateral vessels were compressing the thecal sac at the L4 vertebral level (Axial Image, Figure 2). Computed tomographic (CT) imaging of the abdomen and pelvis was then ordered to confirm suspected thrombosis of the IVC filter. An occlusive thrombus was identified (Figure 3), beginning slightly proximal to the infrarenal IVC filter and extending caudally below the field of view in the right iliac and to the confluence of the internal and external iliac veins on the left.

Venous thrombolysis was contraindicated for this patient because of his recent neurosurgical procedure. Therefore, urgent endovascular intervention was pursued with Angiojet rheolytic thrombectomy (Angiojet Peripheral Thrombectomy System, Boston Scientific, MA, USA), balloon push thrombectomy, and bilateral pelvic vein and infrarenal IVC angioplasty. Although extensive residual thrombosis remained following endovascular intervention, a slight degree of circulatory restoration was achieved and the patient reported mild improvement of his lower extremity pain and weakness. He was then admitted for observation and started on full dose anticoagulation therapy (1 mg/kg lovenox, twice daily). Over the course of the following two weeks, the patient was successfully transitioned to therapeutic anticoagulation on warfarin. Complete resolution of his lower extremity edema and continued improvement in his lower extremity pain was achieved within one week at therapeutic levels. He was discharged home in stable condition. At his three-month follow-up visit, the warfarin anticoagulation was within the therapeutic range, the lower extremity edema had resolved, and the resolution of his trigeminal neuralgia pain continued.

## Discussion

The following noteworthy aspects of this case will be discussed and supported by comprehensive review of the medical literature: (1) the timing of IVC thrombosis within two weeks postoperatively, (2) the degree of occlusion extending caudally from 2 cm above the infrarenal IVC filter into the iliac veins bilaterally, (3) the myelopathy-mimicking symptomatology arising secondary to epidural and paravertebral venous engorgement, (4) the highly unique diagnostic MR imaging that revealed engorgement of Batson’s plexus with prominent flow voids indicating collateral circulation around the thrombosed filter (Figure 2), (5) the use of therapeutic anticoagulation within two weeks following neurosurgical intervention after minimal circulatory restoration was achieved by endovascular intervention.

There are no consensus guidelines for the optimal screening frequency to monitor IVC patency following perioperative filter placement. As major general surgery is a significant risk factor for VTE, close monitoring for IVC occlusive disease is required to minimize morbidity in patients who undergo perioperative IVC filter placement. This case highlights the importance of early diagnostic CT or MR-imaging in surgical patients with an IVC filter who present with myelopathic signs.

The diagnostic imaging included as part of this case is unique. The lumbar MR imaging shows enhancing tissue within the ventral epidural space, prominent flow voids, and tortuous collateral vessels compressing the thecal sac (Figure 2). The ensuing abdominal CT imaging reveals the underlying cause of the venous engorgement seen on MRI: a complete IVC occlusion that begins 2 cm above the infrarenal filter and extends caudally deep into the patient’s right and left iliac veins (Figure 3). According to Kalva et al., total or near-total IVC occlusion occurs in only 0.7% of IVC filters that are screened by CT abdominal imaging within six months following placement [14]. The degree of occlusive disease that we document in this report should be considered an unusual finding even in the context of a known congenital thrombophilia. This fact is supported by Karpenko et al., who investigated the characteristics of phlebothrombosis in a cohort of 54 thrombophilic patients [15]. Of these 54 thrombophilic patients, 40 underwent prophylactic IVC filter placement [15]. Over a six-year interval during which Karpenko et al. performed this study, only three filters progressed to complete occlusion [15].

Indeed, the majority of IVC thromboses culminate in only partial IVC occlusion with minimal or no clinical manifestations [14]. Ahmad et al. performed a large retrospective cohort study of patients with IVC filters and found that 18.6% of patients who received follow-up abdominal CT-imaging had some degree of occlusive disease affecting the filter [16]. Only one patient in this 598-patient study, however, presented with symptomatic IVC occlusion [16]. When filter occlusion does present symptomatically, the presentation is often nonspecific, with the most common clinical signs including lumbar back pain, groin pain, peripheral edema, tachycardia, hepatic engorgement, nephrotic syndrome, and dilatation of superficial abdominal vessels [17-18]. A myelopathy-mimicking distribution of pain and weakness, as observed in the present case, is not frequently associated with IVC thrombosis. This rarity of IVC occlusion as an etiology for radiculopathic pain is demonstrated by Paksoy et al., who found in a cohort of 9,640 patients with lumbar back pain or radiculopathic symptoms that only 10 patients had an underlying IVC thrombosis [19]. Engorgement of Batson’s plexus culminating in radiculopathic pain has been reported for a few rare disease processes: IVC obstruction secondary to Budd-Chiari syndrome, arteriovenous malformation, thrombosis, or a compressing abdominal mass [20-22]. Although diagnostically challenging because it closely mimics much more common pathologies such as disc herniation or spinal stenosis, cord compression secondary to engorgement of the epidural venous plexus must be considered on the differential for all patients with an IVC filter who present with lower extremity pain or weakness [19-20]. With prompt diagnosis and intervention, complete resolution of these symptoms can be achieved.

The optimal management for extensive IVC thrombosis remains to be established [12]. Anticoagulation therapy alone achieves clot regression in less than 50% of cases and complete resolution in less than 5% of cases [12, 23-24]. Alternatively, the current options for mechanical thrombectomy include ultrasonic thrombolysis, rheolytic activity, or aspiration. The literature lacks convincing evidence to suggest that of any one of these mechanical interventions is safer or superior to the others. A recent 10-year retrospective review of patients presenting with symptomatic IVC thrombosis concluded that aggressive endovascular treatment of acute IVC thrombosis is safe and effective with an excellent short-term prognosis [25]. Combined percutaneous mechanical thrombectomy with catheter-directed thrombolysis (CDT) has been shown in several studies to optimize vascular recanalization with minimal thrombolytic dosing [12, 25-27]. Although the optimal form of endovascular intervention continues to rest on expert opinion, a mechanical approach is recommended as the first-line intervention in the postoperative setting; the alternative being anticoagulation, which is supported in the literature only for nonsurgical patients [28].

For this case, the patient’s uniquely extensive thrombosis encouraged immediate invasive management while still within the two-week postoperative window for which venous thrombolysis remained contraindicated. Endovascular intervention with Angiojet thrombolysis, suction thrombectomy, balloon push thrombectomy, and bilateral iliac angioplasty were all attempted with minimal technical success. In this rare context of repeatedly achieving minimal mechanical circulatory restoration in a patient with extensive occlusive disease, the authors suggest admission on a case-by-case basis for observation and initiation of full-dose anticoagulation. Within one week of initiating this treatment, there was a complete resolution of symptoms with the patient recovering to baseline functional capacity.

## Conclusions

IVC thrombosis is an under-recognized and understudied complication that is diagnostically challenging in a postoperative setting. The authors encourage careful postoperative surveillance following perioperative IVC filter placement and early CT abdominal imaging when presented with myelopathic symptoms, edema, dilation of superficial abdominal wall veins, pyrexia, or oliguria. In the rare case of a postoperative patient with incomplete mechanical clot retrieval, initiation of therapeutic anticoagulation is recommended. 
